# The Significance of Social Perceptions in Implementing Successful Feral Cat Management Strategies: A Global Review

**DOI:** 10.3390/ani9090617

**Published:** 2019-08-28

**Authors:** Brooke P. Deak, Bertram Ostendorf, David A. Taggart, David E. Peacock, Douglas K. Bardsley

**Affiliations:** 1 School of Biological Sciences, University of Adelaide, Adelaide, SA 5005, Australia; 2 FAUNA Research Alliance Inc., P.O. Box 98, Callaghan, NSW 2308, Australia; 3 School of Animal and Veterinary Sciences, University of Adelaide, Roseworthy, SA 5371, Australia; 4 Invasive Species Unit, Biosecurity South Australia, GPO Box 397, Adelaide, SA 5001, Australia; 5 School of Social Sciences, University of Adelaide, Adelaide, SA 5005, Australia

**Keywords:** *Felis catus*, invasive species management, public relations, science communication, community engagement, social science

## Abstract

**Simple Summary:**

Feral cats pose a threat to many populations of small- to medium-sized animal species around the world, primarily through predation and disease. For this reason, feral cat management has become a priority in parts of the world where at-risk species are facing high threats of extinction. To implement a successful feral cat management program, there are specific technical and social requirements that need to be met. Most of the recent research conducted around feral cat management has examined the technical aspects, but there is considerably less research around the social aspects that may influence the success of feral cat management in inhabited areas. This review aims to identify and discuss feral cat management from a social perspective, and to highlight potential areas for future research that may aid in building successful campaigns in the future.

**Abstract:**

This review examines the social aspects that influence feral cat management. In particular, it examines definitions and perceptions of feral cats as a species in different countries and across cultures. Using case studies from around the world, we investigate the factors that can influence public perceptions and social acceptance of feral cats and management methods. The review then highlights the importance of social factors in management and suggests the best approach to use in the future to ease the process of gaining a social license for management campaigns. Implications of the influence of education and awareness on public perception and acceptance are further explained, and are suggested to be an essential tool in successfully engaging the community about management in the future.

## 1. Introduction

The cat (*Felis catus*) has been associated with human companionship for thousands of years [1]. As well as being considered to be domestic pets, cats also serve as a means of keeping mice, rats, and other rodent populations under control in homes and on board ships, which in turn has traditionally aided in keeping food and supplies safe, and controlling disease [1,2]. As a result, of their worldwide oceanic journeys however, cats have been introduced to many new environments to which they readily adapted and established invasive exotic populations. Cats now currently inhabit regions of all continents, are present on many islands worldwide, and are impacting the local ecology in those places [2].

Cats are opportunistic feeders that require a high protein diet to sustain themselves, due to their inability to synthesize essential vitamins and minerals [3]. Feral cats, generally defined as cats that have little to no interaction with or dependence on humans, will hunt for food at least several times a day [3,4]. These cats have been at least partially responsible for the extinction of 14% of native bird, mammal, and reptile species worldwide, including in Australia where one third of all recent global extinctions have taken place [3]. In 2001, the species was placed on the International Union of Conservation for Nature (IUCN) list of the 100 worst invasive species worldwide, and was considered the most damaging of the four carnivores on that list due to the impacts they have on endangered species populations [5,6]. One cat alone is capable of depleting populations of smaller mammals and other animals, especially those that are highly concentrated in one area. Consequently, cats can become a major threat to endangered or vulnerable wildlife, which often persist in small, remnant and isolated populations [7]. Even in the case of species of birds and small animals that are not yet vulnerable or endangered, the magnitude of cat predation is high due to hunting by not only feral cats, but by outdoor domestic and stray cats in urban and rural areas as well [3,7].

Cats are also the primary host for several diseases that pose a significant threat to susceptible wildlife, humans, and livestock, which can in turn generate economic risks [8,9]. Some of these diseases, such as toxoplasmosis (caused by the microparasites *Toxoplasma gondii*) and sarcocystis (*Sarcocystis gigantean* and *S. medusiformis*), can be transmitted to humans and other animals through physical contact with a cat or its fecal matter but they have no health consequences for the individual infected cat [10,11]. In humans, toxoplasmosis is a parasitic disease that increases the production of dopamine, promoting a rise in reward-seeking and risk-taking behaviors. This disease has been linked to mental disorders such as schizophrenia and attention deficit disorder, and is known to produce a higher probability of miscarriage and still-birth [12,13]. Children who contract toxoplasmosis within the womb are at risk of suffering from blindness, encephalitis, or developmental retardation [13]. The disease is also a threat to the livestock industry, as it can cause abortions in sheep or weaken newborn lambs, leading to significant economic impacts in areas where sheep farming is a prominent industry [8,14]. Toxoplasmosis has been shown to be carried by feral cats throughout regions of the United States (US), South Korea, Australia, Portugal, South Africa, and Spain [14,15].

Alternatively, *Sarcocystis gigantea* can cause cysts in the muscle tissue of sheep, leading to carcass trimming, or in severe cases, carcass rejection at abattoirs [16]. The disease is dependent upon the complex lifecycle of the microscopic parasites, which begin their development within the intestines of cats that have fed on infected sheep carcasses. These parasites transition into sporocysts that are transmitted to living sheep that graze on pastures contaminated by cat feces. Once ingested by the sheep, the sporocysts localize in the muscles, creating macro-cysts [16]. Although the disease is present throughout countries such as Australia and New Zealand (NZ), it is more common on islands such as Tasmania, where sheep farming is a prominent industry and where there are significant numbers of feral cats [17].

Furthermore, in regions of Europe, feline leukemia (FeLV) as carried by domestic and feral cats is considered a threat to native wild felids such as the endangered Iberian Lynx (*Lynx pardinus*) [18,19]. FeLV occurs naturally as a family of viruses, and is transmitted between felids through direct contact. Once infected, some individuals may experience persistent viremia, which can lead to diseases such as lymphomas, leukemia, or anemia, ultimately leading to the death of the individual within months or possibly years [18,19]. Due to the solitary nature of the lynx, intraspecific fights are common, and contact between lynx and feral cats is thought to have led to the spread of FeLV throughout Iberian Lynx populations [18,19]. Other felid populations such as that of the European wildcat (*Felis silvestris*) are also threatened by the presences of feral domestic cats due to the potential for interbreeding [20]. Hybridization between the two species increases the risk of genetic deterioration for the wildcat population, and increases the likelihood of extinction [21].

### The Need for Management

Because of their destructive nature and negative impacts on environmental and economic wellbeing, governments around the world, especially in the US, Spain, Portugal, Australia, Italy and NZ have initiated feral cat impact control and eradication campaigns [22]. Due to the vulnerability of local biodiversity and livestock in Australia, feral cat management has become a priority, yet some states are currently only beginning to update legislation and expand their feral predator management programs to include cats [23]. Although there are already existing techniques used to control feral predators such as foxes (*Vulpes vulpes*) in Australia, developing effective control methods specifically for feral cats has been the subject of recent public debate [24]. In the US, feral cats are often brought to a shelter to be humanely euthanized, or on rare occasions are selected for adoption based on their suitability to be human companions [25,26]. Legislation in some US states such as Florida and California has included the use of trapping, sterilizing and vaccinating cats, and then releasing them back into the wild [26]. Trap-Neuter-Release (TNR) is considered to be a humane approach to feral cat control, and yet debate still occurs as to whether or not this is the best way to manage local feral cat populations [26,27]. In contrast, Italy has a no-kill policy for stray and feral cats. Instead, it is required by law that any cats caught be sterilized and returned to the wild, and they are not to be euthanized unless found to be terminally ill or dangerous [28,29]. Steps towards planning and implementing feral cat management programs depend heavily on the culture of the location and the associated government choosing an approach that is both socially acceptable and ecologically effective for the region [30].

The primary goal of feral cat management is generally to diminish the population as quickly as possible through humane means [10]. The preferred approach in areas with highly threatened and potentially vulnerable wildlife species is eradication, but eradication campaigns are resource intensive, time sensitive, and costly, especially when applied across large tracts of land where monitoring progress and success can be difficult [31]. For eradication to be successful, the rate of removal for the species must increase at all population densities, and all animals must be able to be detected at low densities for targeted interventions. Due to the nature of these requirements, eradication is considered less feasible than control in large areas [32,33]. However, local eradication is applicable on small islands or within highly managed mainland sites [32,34]. In particular, there have been successful eradication campaigns implemented on islands that have a smaller surface area, where low numbers of cats can be targeted and there is a reduced chance of reinvasion [35,36].

Instead, most feral cat management plans focus on population control and the minimizing of impacts [7]. Management plans that focus on these initiatives tend to be less intensive, and therefore less costly [37]. These programs tend to aim for a gradual decline of the target species population and require a strategic but fluid approach to control in a local area. Although it is important to consider the time and resource allocation required for any program, there is rarely a tangible end goal for control campaigns, with management needing to adjust to changing circumstances. The amount of uncertainty around definitions of program success can often make it difficult to access ongoing funding and continued support for management [37].

Whether it be for eradication or control, many feral cat management programs have adopted an approach that includes the conjoint use of techniques or methods to abate cat numbers, which may include poison baiting, shooting, trapping, TNR, grooming traps, detector dogs, guardian dogs, exclusion fencing, fertility controls and habitat management [7,38]. The techniques selected for use are often chosen based on the size of the target region, the terrain and layout of the landscape, the density of the cat population in the region, and the cost-effectiveness of the suitable techniques [7,39]. Choice of technique also commonly depends heavily on the budget allocation for the campaign, as well as on the tools, time, labor and other resources that are available [30,40].

The application of various methods is contentious for several reasons, some of which are due to misunderstandings around the science behind feral cat management and the knowledge gaps that exist around the issue [41]. There is, however, little debate about the adverse impacts of feral cats on native wildlife. There have been extensive studies conducted on the ecological consequences of feral cat abundance, as reviewed by Tim Doherty, et al. [23]. Experts agree that effective feral cat management needs to be based on the ecology of the predators themselves and their behaviors, while at the same time, acknowledging the strong dependence of any successful ecological outcomes on the effective acceptance and adoption of management by local communities. Thus, research around this topic must take on an interdisciplinary approach [42,43].

Debate around the types of techniques that are used within any regional context are dependent on location and the perceived threat of feral cats as a species in that region. There are also concerns that apply to cats that may not apply in other cases of invasive species management. For instance, certain animals appeal to the public imagination more than others, and iconic species are more likely to attract sympathy and support for their survival [44]. Furthermore, while scientists and management authorities tend to rely on a scientific understanding of the concepts involved in management, the general public and those who may have little to no exposure to threats associated with a target species tend to react to management in an emotional way. This is especially true if the species is one that is considered closely connected to humans, such as the cat [45,46].

For this reason, it is imperative that the local communities associated with the areas where management will take place understand the importance of managing invasive species such as feral cats, so that the programs that rely on public funding are able to generate and maintain support from funding bodies [37]. This may be further complicated by the idea that the definition of a feral cat itself varies depending on location and on the situation of the individual cat [47]. This paper contributes to the discussion around the management of feral cats by reviewing the literature on the social aspects of invasive species management with particular emphasis on feral cats, and the influence of these aspects in the effectiveness of outcomes.

## 2. Community Influence in Invasive Species Management

Any effective invasive species management campaign requires public support and a social license to act, or in other words, the management goals must meet the demands and expectations of society and not act in a way that society feels is unacceptable [48]. Without such a suitable socio-political environment, it is difficult for management campaigns that diminish species populations to succeed [32]. Excessive tension between the community and the administration involved in the delivery of program aims can force management efforts to be frustrated and prolonged [12,32]. In debating the possibility of culling a pest species, particular tensions can emerge between those who are concerned for the welfare of individuals within the target species, and those who are concerned with the welfare of the endangered native species that are at risk due to the presence of the target species [26]. This can often result in prolonged processes of community engagement, deliberation, and assessment to gain public acceptance, without which there would be denial of access to properties, cooperation, regulatory support or funding [49].

For instance, some studies have examined the critical aspects involved in eradicating invasive species including rodents, feral cats and pigs from local islands of NZ and Australia [50]. Although eradicating invasive animals from uninhabited islands is always a management challenge, it was found to be more difficult on islands that were inhabited by people, due to the cost of working to ensure the wellbeing of humans, pets and livestock [50]. Island inhabitants hold varying perceptions of the target species, which can prolong program planning. In one particular case study, the residents of Waiheke and Pitt Islands accepted the eradication of mice and feral cats as a benefit to the island, but the eradication of pigs was deemed undesirable as they are considered a hunting asset [50]. On Lord Howe Island, the community seemed skeptical about the benefits of an eradication campaign, and ongoing deliberation has since delayed the program. The eradication of stoats (*Mustela erminea*) on D’Urville Island, NZ was also delayed for 10 years due to insufficient community acceptance, and it was only after extensive debate that social support for the control program was generated [50]. As a result of the social barriers of acceptance faced by management, gaining a social license has been considered one of the main constraints in planning and implementing invasive species management campaigns [48,50].

Community engagement can contribute to the generation of social license by involving individuals in planning and management, which ensures learning of the processes involved in management and building upon local skills and knowledge [51,52]. One study of community engagement within an Australia region considering wild dog management found that it was essential to contextualize pest management in relation to local concerns to gain community approval, promote understanding and facilitate success [51]. Other studies suggest including animal welfare organizations in the decision-making process may further increase public support, as doing so is more likely to ensure that the management methods selected are humane and have been discussed from varying, often conflicting viewpoints [53].

In a similar example, education and knowledge are highlighted as essential factors in increasing support and gaining a social license for management campaigns [54]. An Irish campaign was implemented to eradicate invasive muskrats from the country, as they were posing problems for native plants and crops throughout, and were known to damage drainage systems, as well as burrow in unacceptable areas [54]. Due to these and other concerns around potential riverbed erosion, the government implemented a plan to eradicate the species. As it was seen by both the community and other stakeholders to be a species that was detrimental to the environmental and economic wellbeing of the country, financial support as well as social license was given to ensure that the eradication program would be a success [54]. The program ran for two years, and the success was partially attributed to the education and knowledge within the community, as well as key stakeholders about the species and its impacts [54].

The lack of education and knowledge around a pest species and its impacts can also impact proposed management, as demonstrated by a study of feral cats in University of KwaZulu-Natal in South Africa. The university investigated perceptions of students and staff around the feral cats found in Msinsi Nature Reserve (the Conservancy). The results of the study suggested that although the Conservancy believed the feral cats to be an exotic species of high threat to the native wildlife of the area, most respondents did not define them as exotic or believe that they posed such a threat [55]. While the Conservancy had aimed to eradicate the feral cats, most of the participants in the study believed that only population control and management was necessary, and not eradication [55]. Furthermore, there was no consensus on what methods should be used to control the cats in this area due to lack of understanding around the methods. Few respondents were aware of the aims of the Conservancy or of the impacts that feral cats have on native wildlife, and as a result it was suggested that university students and staff be encouraged to learn more about their local ecology, and to volunteer with the Conservancy as a way to learn through experience [55].

Society is sensitive to educational programs, and successful education campaigns can lead to a better sense of awareness about target species and their impacts [46]. In the Netherlands, a study found that participants who were highly educated or engaged in environmental activities were also more likely to support invasive species management than those who were unfamiliar with the topic [56]. Those who were more knowledgeable were also more likely to understand the level of risk associated with different invasive species and support their effective management [56]. The familiarity and perceptions associated with feral cats, though, can differ greatly depending on how a country or groups within a location define the term.

## 3. The Elusive Definition of a ‘Feral’ Cat

There are many definitions given to feral cats [57]. In a general sense, most definitions suggest that it is a cat that lives in the wild, does not interact with human beings or rely on them for food or shelter [47]. In contrast, stray cats are normally defined as free-roaming cats that stay close to human habitation and rely indirectly on humans for these resources [58]. The greatest confusion seems to occur when discussing the difference between a stray and a feral cat, as countries such as the US and parts of Europe draw a fine line between these terms, sometimes even using them interchangeably [57,59]. For instance, in the US there are cat colonies that consist of both feral and stray cats, often called “community cats” [27,60]. These colonies are cared for by volunteers from the community who provide resources for the cats, including sheltered areas and feeding stations [61]. The supposed difference between feral cats and stray cats in this instance is that feral cats tend to be incredibly wary of the humans that care for these colonies, and stray cats seem to be more approachable [61]. In a formal context, Michigan State University Law School defines feral cats as those that were once owned and were either abandoned, lost, or had run away. The descendants of such cats are referred to as “stray” cats with later generations becoming “feral” [62]. Certain states within the US also have laws pertaining to “owning” feral cats, though these laws are difficult to enforce as multiple counties within a state may have different interpretations [63].

Part of the issue in defining what a feral cat truly is may lie in the fact that the status of a cat may change depending on its situation [57,64]. A domestic cat that has been abandoned by its owners can become a stray or can turn feral, and its offspring would then be considered stray and/or feral as well [58]. Most kittens, if they are found young enough, are usually able to be socialized and adopted out as domestic pets [57]. If an adult cat is caught and found tame enough to be adopted, it can also once again become a domestic pet [57]. In the case of feline colonies that are maintained by humans, it is difficult to determine whether the cats that live within the colony should be defined as stray or feral, as it seems to depend on whether individual cats use the resources provided by the humans, and whether they are in contact with the humans caring for the colony [60]. As they are mostly defined as “community cats”, the difference between a stray and a feral cat becomes even more vague to the general public [61]. Although the Michigan State University Law School has set a definition for a stray cat and a feral cat, their classification would be difficult to apply for management purposes [62,63]. Related documentation refers to a cat’s potential behavioral changes over its lifespan as a possible reason for the vague definitions in certain European countries as well [59].

In Spain and Italy, the term feral cat is used to describe a domestic cat that has been abandoned or returned to the wild [14,65]. These cats often live in colonies throughout urban areas and are given food, shelter and other resources by humans [14,65]. They are also referred to as street cats, which one could easily confuse for a stray cat instead of a feral cat [65]. This is especially true in Rome, where urban domestic cats are known interchangeably as both feral and stray cats, and are believed to be overfed by local citizens who leave an abundance of food out for them [28]. Other European countries take a slightly different approach to definition. In Estonia, five types of cat are defined, including feral cats and semi-feral cats [66]. In this context, feral cats are known to hunt and fend for themselves, though they will occasionally scavenge leftovers produced by humans. They are considered skittish and are generally afraid of people. Semi-feral cats will hunt but will also accept food left out by humans, and although they show no fear towards people, they will not establish a bond with them directly [66]. Pseudo-wildcats are also classified in this study, and are said to be different from feral cats in that they are completely independent of humans [66]. In contrast, the UK has no recognized definition of a feral cat, and the wide variations of what people in the country believe a feral cat to be can range significantly due to the belief in the possibility of “taming” a feral cat given time [57].

The differences in definition within these countries may reflect the level of perceived threat that feral cats pose to the natural environment. The US and Europe host native felids that have evolved with the changing environment over time, and the introduction of feral cats into these regional ecosystems has not seemed to have had detrimental impacts. However, this is not the case in countries such as Australia and NZ, where no native felids existed prior to the introduction of cats. Native ecosystems and associated species within these countries have not had the opportunity to adapt to the presence of cats, and so the threat that stray (semi-feral) and feral cats pose is much higher. The need for management in this case requires a clear and firm definition of what a domestic, stray and feral cat is, and this cannot be determined by definitions granted by the US or Europe, as the closest definition to a feral cat in the Australian context would likely be that of the aforementioned pseudo-wildcat [7].

Therefore, the differences between a stray cat and a feral cat are far more pronounced in Australia. Stray cats or semi-feral cats are those that were once owned and have either been abandoned or run away [67]. They wander through urban areas and adjacent bushland or farmland, hunting and killing wildlife, but also accepting food and resources from humans. They are sometimes thought able to be rehabilitated back into pets. Feral cats in Australia, however, are thought to have never been owned by humans, and inhabit bushland areas away from human habitation [67,68]. The Australian government has also nationally declared feral cats as a pest species that requires appropriate management [69]. In NZ, research has specifically aimed at defining the nature of different categories of cat, whether they be a Companion, Stray or Feral Domestic Cat [58]. For the sake of management, stray cats were noted as those that live in colonies or close to human habitation, and that indirectly rely on humans for their needs [58]. Their numbers could be augmented by interbreeding with the companion cat population, but they were not considered feral cats. Feral cats were defined as cats that had none of their needs provided for by humans and lived in areas away from human habitation. The feral cat population in this case is said to fluctuate independently of companion cat influence [58].

With the definition of a feral cat being conditional on the region, there is also a large variance in the way that feral cats are perceived in the media and different parts of the world [70]. Perceptions and attitudes of the general public can be influenced by a range factors, including the knowledge that an individual has around a topic, and the way that information is received and interpreted by that individual [45]. Science communication plays a large role in the way people perceive scientific topics such as feral cat management, and this is especially true in highly networked democratic societies where the public increasingly has a say in the scientific and technological solutions that are implemented by policy [71,72]. There has recently been a major push towards improved efficiency in science communication and dialogue between those in management and the general public [71,73]. Developing a universal and solid definition for the terms associated with both “stray” and “feral” cats may aid in improving this efficiency by allowing better understanding to be formed through different media outlets worldwide. This could further aid in stray and feral cat management, as a better understanding would allow the general public to take a stronger stance on these cats as a ‘category of animal’ that needs to be managed [74]. It may also enable management authorities to more clearly design cat control measures to address either feral or stray cats, depending on the degree of social acceptance of various control measures for cats within each of these categories.

This may also aid in reducing confusion around how feral cat management should be reported by journalists. Media stories are often presented as quickly as possible and truth can sometimes be skewed, or key details overlooked [74]. Opportunities can often be missed to properly educate the public on the topics that are covered, such as what a feral cat actually is in management terms [73,75]. As a result, much of the information that the general public is introduced to on scientific topics, including invasive species and their management, is condensed to highlight only a few main points around which the public can begin to develop an informed stance [73]. As most people rely on old or new media sources for news in a global and local context, the way this information is received can affect the way that an individual views the topic through way of agenda-setting [76,77]. First level agenda-setting theory suggests that media coverage can influence what people think about a topic, with second level agenda-setting prying deeper to suggest that it also influences how people think about that topic [77].

In reference to feral cats, news stories tend to be divided depending on narrative framing, either reflecting a positive or negative view of cats [72,76]. One news source in particular, the New York Times, has written stories throughout the years that frame cats in multiple ways, including as villains, victims, heroes, commodities, and as women’s best friend, which may further confuse the perception of the public [78]. When faced with numerous stories framed in a specific way, an individual may form a perception that is associated with these views [76]. Word choice dependent on the narrative framing of the story can further impact perception, as people are more sympathetic towards terms such as “community cat” or “outdoor cat” than they are to “feral cat,” even if both terms are referring to the same animal [26].

## 4. Perceptions Around Feral Cats and Their Management

General public perceptions around an invasive species can vary dramatically, making it difficult for those in management to appeal collectively to a diverse group of stakeholders [55]. Even if most stakeholders hold similar perceptions about a species and the risks associated, opinions of management interventions can vary. In most cases the general public is familiar with an invasive species issue within the local context due to direct experience or what they view in the local media. Early stages of stakeholder consultation on the risks posed by the species is often considered the best approach to incorporating public values into policy and drawing support from the community [54,55]. In the case of feral cats and their management, there is often a high level of contention within the general public, as many people make the emotional connection with cats and relate feral cats to privately owned domestic cats, while others focus on direct negative impacts [24].

In Australia, native fauna is highly regarded and widely valued by society. Feral cats are considered a threat by many, though there are various views shared by different demographic groups [79]. Cultural views often influence an individual’s perception of the human-nature relationship, and can thereby frame individual views of a certain species within the natural environment, whether it be native or non-native [79]. For instance, within indigenous Australian culture, some individuals view feral cats as a threat to native fauna, whereas others accept them as an introduced part of the landscape, or view them as an integral part of their Dreaming, or their understanding and interpretation of the world and how humans fit into that world [79].

A Danish study found that most of the population, about 60%, did not see a problem with allowing cats to roam freely, while about 27% did consider them to be an issue [80]. The other 13% in this study had no opinion on the topic. Most of the people who did consider roaming cats to be a problem had never had a cat for a pet, and those that had were more likely to accept the free roaming of cats [80]. Though perhaps not directly labelled as feral cats in the study, the sentiment of non-cat owners expressing stronger dislike for roaming cats may present a common theme in studies of perception around feral cats and their management, and should be carefully considered [80].

In some European countries such as Estonia and Italy, feral cats are perceived to be both a nuisance and a staple of the urban environment [28,66]. In an urban setting, feral cats are often thought to add to the aesthetic of the environment, as they have roamed the streets of the Western world since ancient times and are today considered part of the décor [28,66]. As well, these cats are believed to benefit the lives of the elderly women who care for them, and who are often called “feeding ladies” and are part of an intercultural theme that occurs in much of Europe and the US [61,66]. Another practical benefit in both urban and rural locations throughout these countries is that feral cats are considered an integral part of controlling rodent populations, and they are sometimes even adopted onto farms as “barn cats” for this specific reason [27,66,81]. On the other hand, feral cats are also seen as a nuisance by some in Europe due to the diseases they potentially carry, their threat to native wildlife, and their behavior in public areas, such as defecating in public spaces and yowling or hissing at night [66,81]. At the same time, they are not necessarily considered a pest species in these spaces, and for that reason they are treated with concern and care when it comes to management [28,66]. Instead of lethal methods, the method often used in their management is TNR, with the aim of reducing the breeding population. This method is viewed by the European and US public to be a humane option to controlling feral cat populations as opposed to trapping and euthanizing the animals [61,66].

Along with the general public’s perceptions, different stakeholder groups often hold their own views on feral cats, which may be linked to the nature of and culture within the group [80]. Natural resource managers, conservation groups, and landowners that have dealt directly with feral cats are knowledgeable and aware of the impacts that they can cause to the natural environment, and will normally take steps to mitigate these impacts [2,31]. In contrast, animal welfare activists, cat owners and cat colony enthusiasts are likely to strongly argue against the culling of feral cats, or against certain lethal forms of management that are lethal such as poison baiting [2,31].

## 5. Controversy Around Management Methods

As governments in various countries implement feral cat management plans, the subject of *whether* the cats should be managed shifts to a question of *how* to manage them, creating additional controversies [82]. Community and stakeholder participation is vital to gain support for an approach that is considered ethical and acceptable by the majority, and for that reason it is essential to examine opposing views associated with all relevant management techniques in an area [82]. Some techniques, such as baiting with different types of poison, are perceived to be unethical due to their nature and the amount of time required to work. Additional hesitation in adopting techniques comes from the potential threat posed to domestic pets or other non-target animals that may be exposed [83,84].

For example, a particular method within Australia that has been met with negative feedback by the public according to social media and news sources is the use of the chemical sodium fluoroacetate, commonly known as 1080, in baiting and in grooming traps [84]. This chemical is considered unethical by some stakeholders due to the amount of time required to take effect, and because of the symptoms of apparent discomfort that animals may exhibit as the poison takes effect. There is also fear among the public about the potential for pets to ingest the poison, which could lead to death. Although the chemical is found in native plant species in Western and central Australia, it is also thought to be potentially dangerous to native non-target species in areas outside of the state, or areas that are far from these plants [84]. For that reason, a permit is required for use in other states within Australia and NZ, and it is highly restricted for use in other countries such as the US, which heightens the debate over its use in feral cat management overall [84].

In the US, where the TNR method is highly controversial, it is up to the discretion of the state and local governments to control feral cat populations, and many have adopted TNR as a normal practice [85]. However, some scientists believe that this method is ineffective in controlling feral cat populations, and that it does not negate the impacts of the cats [27]. They believe, instead, that trapping and euthanizing individuals would be a more effective means of control. This also applies to organizations such as the Audubon Society, which has openly criticized the technique for not eliminating the threat to wildlife or reducing cat numbers [26]. The organization instead aims to encourage pet owners to keep their cats indoors to reduce cat populations and decrease the risk to wildlife. In contrast, feral cat advocates claim that the risk these cats pose to wildlife is widely overestimated, and they fully support TNR as a way of promoting the benefits of feral cats and their colonies [26]. Demographics within the US may further influence attitudes towards management methods with research suggesting that people who live in rural areas tend to be more in favor of lethal control of feral cats, and that shooting was the preferred method. It was also noted that this may be dependent on the values of the people within the rural regions, as they view animals according to their usefulness, and feral cats were seen to be more destructive than beneficial to the natural environment [56,83]. On the other hand, those who lived in urban areas preferred TNR programs over euthanasia, and this may be a reflection of their values, their views of feral cats in the natural environment, and their level of knowledge around cat impacts on native wildlife [83].

This review has found that a range of studies have examined perceptions of feral cats and their management in different places worldwide, though most of these examine methods that are suited specifically to target areas. Also, each country has its own definition of a feral cat, and therefore the contest of the approach to feral cat management depends on the culture, beliefs, gender differences, and perceptions of its people. Due to these variances, there is no universal understanding of how the general public views either feral cats or different feral cat management methods [82,83]. Furthermore, recent studies on perception around feral cat management have focused on a broad overview, but there is little to no research that compares how participants in different locations may view the potential for management methods being used near their land. There is also little to no research around attitudes towards having certain management methods used directly on individual properties, or how this may vary according to certain demographics such as location, occupation, or land use. This type of research could help decision-makers in determining what methods may be viewed as acceptable by both individual and broader geographic communities, and which methods may be more feasible in gaining social license for management in any given location.

## 6. Conclusions

This review has highlighted the importance of consulting all stakeholder groups that have an interest in feral cat management, including the general public, prior to plan implementation. It has also outlined the benefits that may accrue through more thorough investigation of public perceptions and attitudes that influence views about feral cats and cat management among stakeholders. This includes assessment of the public’s knowledge and familiarity around feral cats and their impacts, and the potential for increased education campaigns to impact decision-making.

Furthermore, a general understanding of the level of threat that feral cats pose within different regions needs to be developed with associated knowledge of how these threats vary in relation to the different categories of cat. Developing a solid, universal definition about what a feral cat is, as opposed to a stray cat, will aid in improving the efficiency and effectiveness of management and will serve as the starting point for identifying what actions need to be taken in relation to eradication or impact control. This development will also aid in bridging the gaps in knowledge and understanding between scientists and management authorities designing management plans worldwide. From there, guidelines and education campaigns could be developed to efficiently communicate these differences to the public and to raise awareness around the threats of feral cats. With a firmer understanding of what feral cats are and the threats they pose, there is a higher chance that the public will support management efforts. Furthermore, research into the types of technical solutions that would meet and abate social concerns in different regions may aid in helping managers to identify techniques that may be seen as less controversial while still being effective.

Research would also benefit from investigating the drive behind general public and stakeholder interests, the cultural values and definitions associated with feral cats as a species, and in further detail the attitudes and values of the individuals associated with feral cats and management. This may help to determine the types of individuals that favor feral cats and non-lethal methods as opposed to those who would prefer to remove these cats by any means necessary. It would also aid in identifying the communities, either local or global, that are in support of managing feral cats, those that are not, and how to properly approach these different communities and communicate in a way that will help to gain social license for feral cat management.
